# A 3D Renal Proximal Tubule on Chip Model Phenocopies Lowe Syndrome and Dent II Disease Tubulopathy

**DOI:** 10.3390/ijms22105361

**Published:** 2021-05-19

**Authors:** Sindhu Naik, Andrew R. Wood, Maté Ongenaert, Paniz Saidiyan, Edo D. Elstak, Henriëtte L. Lanz, Jan Stallen, Richard Janssen, Elizabeth Smythe, Kai S. Erdmann

**Affiliations:** 1 Department of Biomedical Science, Centre of Membrane Interactions and Dynamics, University of Sheffield, Western Bank, Sheffield S10 2TN, UK; sindhu.naik@glpg.com (S.N.); arwood1@sheffield.ac.uk (A.R.W.); 2 Galapagos BV, Zernikedreef 16, 2333 CL Leiden, The Netherlands; p.saidiyan75@hotmail.com (P.S.); edo.elstak@glpg.com (E.D.E.); jan.stallen@crl.com (J.S.); richard.janssen@glpg.com (R.J.); 3 Galapagos NV, Generaal de Wittelaan L11, A3, 2800 Mechelen, Belgium; mate.ongenaert@gmail.com; 4 Mimetas BV, J.H. Oortweg 19, 2333 CH Leiden, The Netherlands; h.lanz@mimetas.com

**Keywords:** organ-on-a-chip, disease modeling, proximal tubule-on-a-chip, Lowe syndrome, fibrosis, microfluidic, OCRL

## Abstract

Lowe syndrome and Dent II disease are X-linked monogenetic diseases characterised by a renal reabsorption defect in the proximal tubules and caused by mutations in the OCRL gene, which codes for an inositol-5-phosphatase. The life expectancy of patients suffering from Lowe syndrome is largely reduced because of the development of chronic kidney disease and related complications. There is a need for physiological human in vitro models for Lowe syndrome/Dent II disease to study the underpinning disease mechanisms and to identify and characterise potential drugs and drug targets. Here, we describe a proximal tubule organ on chip model combining a 3D tubule architecture with fluid flow shear stress that phenocopies hallmarks of Lowe syndrome/Dent II disease. We demonstrate the high suitability of our in vitro model for drug target validation. Furthermore, using this model, we demonstrate that proximal tubule cells lacking OCRL expression upregulate markers typical for epithelial–mesenchymal transition (EMT), including the transcription factor SNAI2/Slug, and show increased collagen expression and deposition, which potentially contributes to interstitial fibrosis and disease progression as observed in Lowe syndrome and Dent II disease.

## 1. Introduction

Lowe syndrome is an X-linked monogenetic human disease mainly affecting the eye, brain, and kidney and, thus, is also known as oculocerebrorenal syndrome, which gave rise to the name OCRL for the gene mutated in Lowe syndrome [1,2]. Mutations in OCRL can also be found in a subset of Dent disease patients, which led to the establishment of a Dent II disease category for these patients [3,4]. Dent disease patients show a very similar renal phenotype but differ from Lowe syndrome patients by not showing a major phenotype in the nervous system or eye. Lowe syndrome and Dent II disease patients both show a progressive reabsorption defect in the kidney that often leads to complete kidney failure and which is the main cause for increased mortality observed in Lowe syndrome patients [5,6].

The OCRL protein is an inositol-5-phosphatase catalysing the removal of phosphate from the position 5′ of the inositol head group of phosphatidylinositol-4,5-bisphosphate [PtdIns(4,5)P_2_] and phosphatidylinositol-3,4,5-trisphosphate [PtdIns(3,4,5)P_3_] [7]. OCRL is a peripheral membrane protein binding to several members of the monomeric G-protein Rab-family [8,9]. OCRL is mainly localised at the Golgi apparatus and at early/recycling endosomes but can also localise to lysosomes under conditions of high endocytic load [10,11,12]. OCRL has been shown to play a major role as a regulator of intracellular trafficking, in particular contributing to the uncoating of clathrin-coated vesicles as well as regulating receptor recycling from endosomes to the plasma membrane or to the Golgi apparatus [11,13,14,15]. Results from Lowe syndrome animal models as well as from isolated proximal tubule cells from Lowe syndrome patients reveal a role in the recycling of megalin, a receptor important for nutrient absorption in the kidney, which accounts, at least in part, for the renal reabsorption defect observed in Lowe syndrome and Dent II disease [15,16,17].

OCRL has also been reported to localise to the basal body and the transition zone of the primary cilium [18]. Fibroblasts or proximal tubule cell lines compromised in OCRL protein expression show fewer and shorter cilia, while knockdown of OCRL in MDCK cells (a kidney cell line derived from the outer medullary collecting duct) has been reported to lead to longer cilia [18,19,20,21]. Cilia play a major role in sensing and transducing chemical and mechanical cues, and it has been reported that the lack of OCRL leads to an increase in PtdIns(4,5)P_2_ levels at the transition zone of cilia causing reduced accumulation of sonic hedgehog protein upon stimulation with a hedgehog agonist [22]; however, the role and contribution of cilia defects to the Lowe syndrome phenotype are unclear.

Suitable model systems are key to investigating disease mechanisms and to identifying and testing potential drugs and drug targets for Lowe syndrome. Early attempts to establish a Lowe syndrome mouse model by knocking out the *Ocrl* gene failed as *Ocrl* KO mice did not develop a disease phenotype [23]. The reason for this is the presence of another inositol-5-phosphatase, INPP5B, that can compensate for the lack of OCRL in mice but not in humans. *Ocrl* and *Inpp5b* double knockout mice are embryonically lethal; however, an *Ocrl* knockout mouse with a tissue-specific knockout of *Inpp5b* in kidney is viable and shows a renal reabsorption defect [24]. Furthermore, expressing humanised INPP5B in *Ocrl*/*Inpp5b* knockout mice shows proximal tubule dysfunction (low molecular weight proteinuria, aminoaciduria) similar to Lowe syndrome and Dent II disease [16,25]. Finally, transgenic zebrafish embryo models with impaired OCRL expression have been generated showing defective fluid phase uptake and clathrin-mediated endocytosis in pronephric tubules and are more susceptible to febrile seizures and develop cystic lesions in the brain [17,18,26,27]. However, human Lowe syndrome model systems are currently limited to static 2D cell cultures of proximal tubule cells or skin fibroblasts isolated from Lowe syndrome patients or kidney-derived cell lines using knockdown or knockout technologies to impair OCRL expression.

Given the severity and life-threatening nature of the renal phenotype of Lowe syndrome novel, more physiologically relevant human in vitro models to investigate this disease aspect in particular are necessary. In vivo proximal tubule cells are arranged in 3D tubules and are exposed to significant fluid flow shear stress, which is not reflected in current static cellular models [28,29]. Here, we describe a novel proximal tubule on chip model for Lowe syndrome and Dent II disease that phenocopies hallmarks of these diseases. Using this model, we reveal that proximal tubule cells lacking OCRL demonstrate upregulation of epithelial–mesenchymal transition (EMT) marker proteins and increased collagen deposition, which may contribute to interstitial fibrosis as observed in Lowe syndrome patients.

## 2. Results

In order to develop an organ on chip model for Lowe syndrome, we first engineered a suitable cellular model for Lowe syndrome by deleting OCRL protein expression in the human proximal tubule cell line HK-2 using CRISPR/Cas9. HK-2 is a robust cell line, easy to grow and well established in the field of proximal tubule research [30]. Lack of complete OCRL expression has been shown previously in several Lowe syndrome patients, and genomic nonsense and frame shift mutations leading to early premature stop codons have been identified in Lowe syndrome and Dent II disease patients [3,31]. Thus, knocking out OCRL is a feasible approach to model Lowe syndrome.

**Figure 1:**
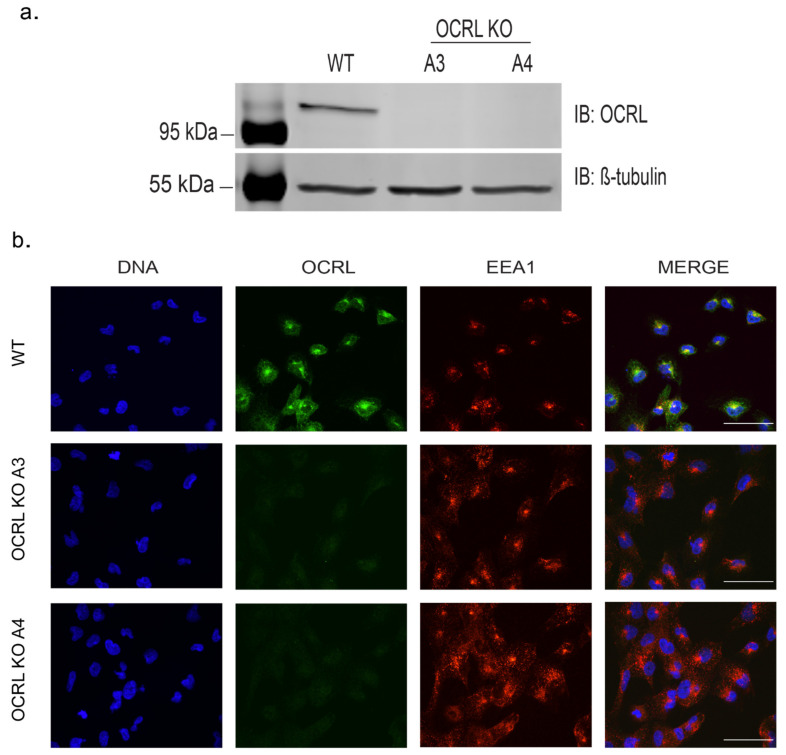
Confirmation of OCRL KO stable cell lines: (**a**) Western blotting with Anti-OCRL–No OCRL protein was detected in A3 or A4, but WT shows the band at the predicted size of 110 kDa. Anti-β tubulin at 55 kDa was used as a loading control. (**b**) Immunostaining of WT and KO clones with Anti-OCRL and EEA1 (early endosomes) showing no punctate staining for OCRL in KO clones (A3 and A4). Images were taken by Airyscan confocal microscope, scale bars: 20 µm.

We chose two independent OCRL KO clones for further analysis. Lack of full-length OCRL protein was confirmed by Western blotting and by immunofluorescence analysis (Figure 1a,b). Genomic characterisation revealed frame shift mutations leading to predicted early premature stop codons (Figure S1).

Next, we investigated whether our OCRL KO cell lines displayed phenotypes previously described in cells from Lowe syndrome patients and, thus, could serve as a suitable cellular model for Lowe syndrome in principle. A hallmark of Lowe syndrome (and Dent II disease) is a reabsorption defect in proximal tubule cells. Reabsorption in proximal tubules takes place mainly via the megalin/cubilin receptor system. Megalin also binds the receptor-associated protein RAP, which serves as an exocytic traffic chaperone and inhibits ligand binding to the receptor [32]. Using a recombinant GST–RAP fusion protein, we exploited the specific binding of RAP to megalin to monitor and quantify megalin-mediated endocytosis in wild-type and OCRL KO HK-2 cells [15]. We observed that OCRL KO cell lines showed a significantly reduced uptake of RAP compared to wild-type HK-2 cells at all time points investigated (Figure S2). Previously, it has also been reported that reduced reabsorption is, at least in part, due to the reduced cell surface expression levels of megalin [15,17]. This is in line with our finding of reduced cell surface binding of GST–RAP to OCRL KO HK-2 cells (Figure S3). We also observed increased F-actin co-localisation with EEA1, reflecting aberrant actin polymerisation at early endosomes, likely due to the elevated PtdIns(4,5)P_2_ levels as described previously (Figure S4) [15]. Furthermore, we observed mis-sorting of CI-M6PR to early endosomes (Figure S5) also as described previously [33]. Overall, our findings are in line with a previously described characterisation of HK-2 OCRL KO cells [34].

In vivo proximal tubule cells form a 3D laminated structure and are exposed to significant mechanical stress in the form of fluid shear stress. This 3D architecture combined with fluid shear stress is an important regulator of cellular homeostasis and is, so far, lacking in current in vitro models for Lowe syndrome.

**Figure 2:**
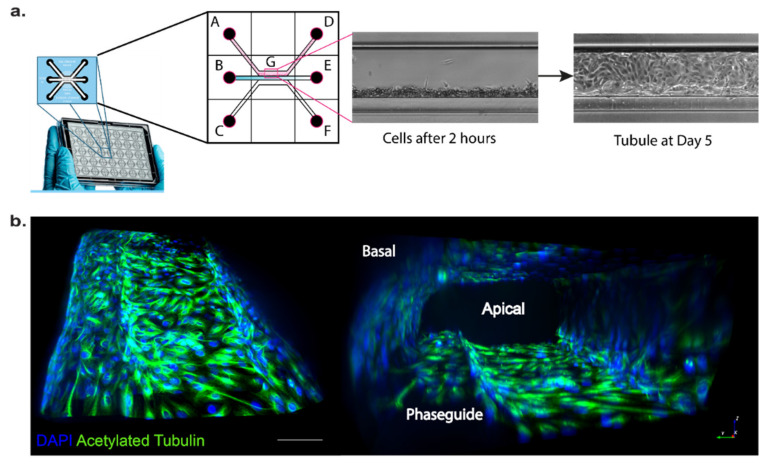
Proximal tubule on a chip: (**a**) 3-lane OrganoPlate^®^ with 40 chips, a pictorial representation of a single chip with all its wells (A: cell inlet, B: gel inlet, C: medium inlet, D: cells outlet, E: gel outlet, F: medium outlet and G: observation window). The observation window (G) with the top lane shows when cells adhere against the gel in the middle lane followed by cells forming a tubule at Day 5. (**b**) Immunostaining images of the 3D proximal tubule grown in OrganoPlate with acetylated tubulin (green); scale bar: 500 µm.

**Figure 3:**
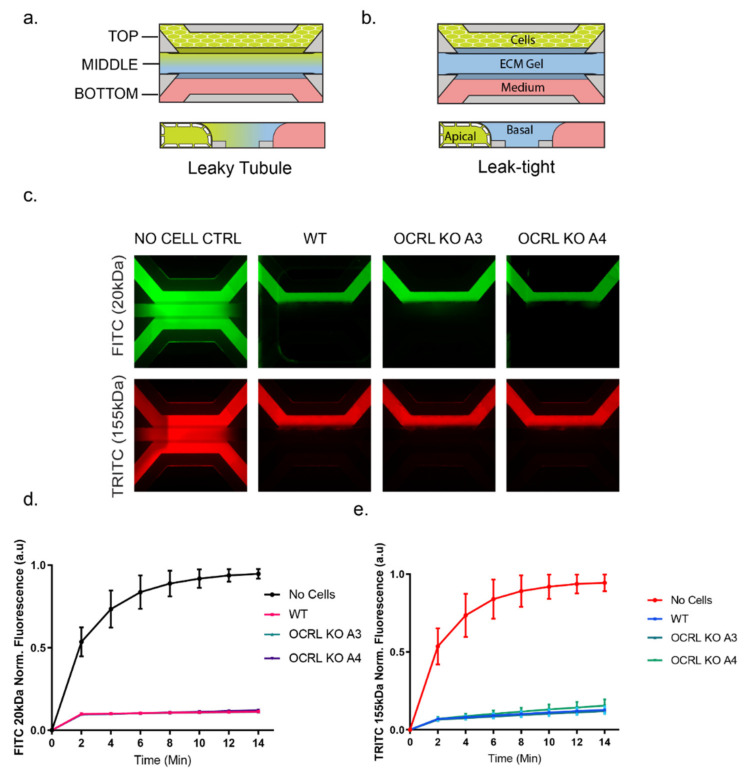
Barrier integrity assay: (**a**) a cartoon of the 3-lane OrganoPlate microfluidic chips with a top perfusion channel with cells, middle gel channel and bottom medium channel. When dye-containing medium was added in the lumen, the dye did not permeate into the gel channel if the tubule was leak tight. (**b**) If the tubule was leaky or in cell-free control chips, the dye diffused into the gel channel. (**c**) Images of the leak-tight tubules of HK-2 cells in WT and OCRL KO clones (A3 and A4) vs. a cell-free control for leakiness for both 20 kDa FITC Dextran and 155 kDa TRITC Dextran molecules. (**d**,**e**) Graphs showing the curve for the cell-free control chip with the diffusion of the dye and its normalised fluorescence intensity increasing over the duration of the assay (14 min). Leak-tight tubules had stable ratios of signal intensities in the perfusion channel vs. the gel channel. The XY graph was plotted with the mean and SD values, *N* = 3. Each *N* had values from at least 5 chips per sample.

Having demonstrated that our OCRL KO HK-2 cell lines display hallmarks of the phenotype described previously for cells isolated from Lowe syndrome patients, or in animal Lowe syndrome models, we incorporated our cell lines into a three-lane OrganoPlate^®^. OrganoPlate is a recently developed microfluidic platform that allows for the growth of 3D proximal tubules in a microfluidic setup [35,36,37]. Cells were seeded into the top channel, then the middle channel was filled with ECM (collagen I) and the bottom channel was filled with medium. Cells were grown for several days under fluid flow stress by placing the plates on a rocker using gravity to induce fluid flow and form a three-dimensional tubule with a hollow lumen (Figure 2). To test the integrity of the 3D tubules formed in the OrganoPlate setup, we used a dextran diffusion assay by loading fluorescently labelled dextran (20 kDa or 155 kDa) into the top channel and followed diffusion into the middle and bottom channel over a time course of 14 min using fluorescence microscopy. Tubules formed by wild-type or OCRL KO HK-2 cells did not show any significant diffusion of fluorescently labelled dextran into the middle and bottom channels, whereas dextran loaded into the top channel housing no tubules as a control leaked into the middle and bottom channels (Figure 3). We concluded that OCRL KO cells could form leak-tight tubules.

**Figure 4:**
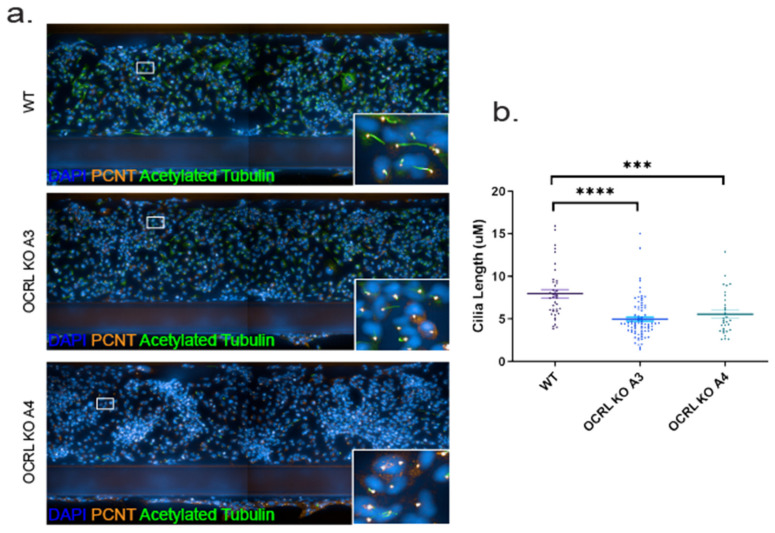
Cilia defect in Lowe syndrome cells under fluid flow: (**a**) immunofluorescence images showing HK-2 cells (WT and OCRL KO (A3, A4)) grown in OrganoPlate and stained with acetylated tubulin and PCNT to visualise primary cilia (green) and pericentrin (orange), respectively. Images were captured using the Perkin Elmer Operetta CLS with 20X water objective. Two adjacent fields were stitched together for visual purposes of the tubule. (**b**) Quantitative analysis of cilia length measured using segmented tool in ImageJ software and represented as a scatter plot with individual values. Ordinary one-way ANOVA and Dunnett’s multiple comparison test, *N* = 3 (>50 cells per *N*). ***, *p* < 0.001; ****, *p* < 0.0001, error bar represents mean with ± SEM.

In zebrafish models for Lowe syndrome and fibroblasts isolated from Lowe syndrome patients, defects in cilia formation have been reported [18,21,27]. We tested whether this was also the case for human HK-2 cells lacking OCRL and which have been exposed to fluid flow stress similar to the situation in the human kidney or zebrafish pronephros. Both OCRL KO clones investigated showed significantly reduced cilia length (Figure 4).

**Figure 5:**
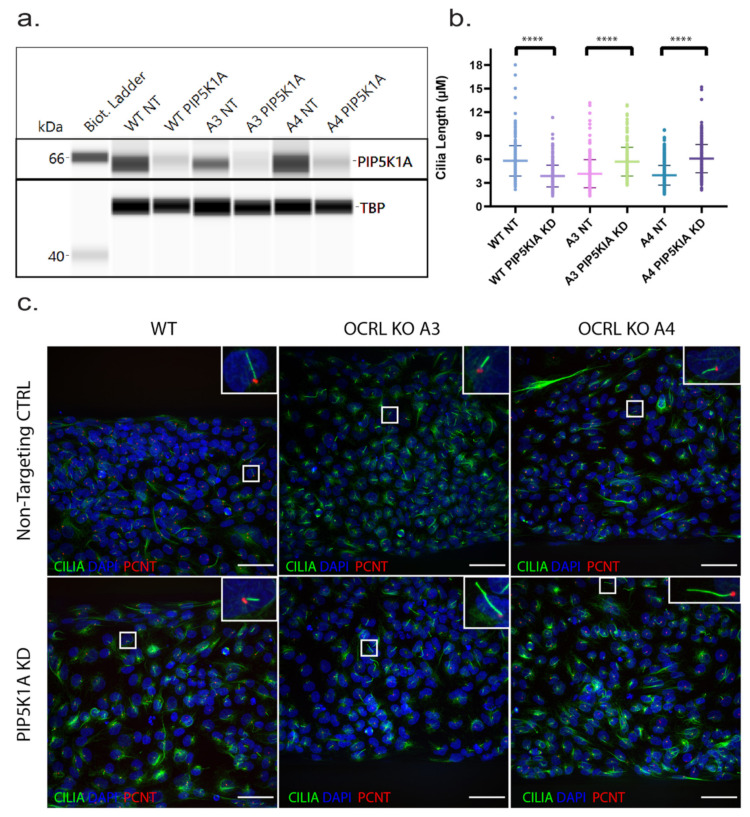
Cilia length defect in OCRL-KO cells was rescued by PIP5K1A KD: (**a**) Capillary-based Western blotting (WES) confirming a reduction in protein expression levels of the PIP5K1A gene in WT, OCRL KO A3 and A4. The first lane depicts the biotinylated ladder at 66 kDa. The bands detected by the PIP5K1A antibody with samples of non-targeting siRNA CTRL show a distinct band at ~65 kDa. TBP (TATA-Box Binding Protein) was used as a loading marker and detected at ~52 kDa. Images were derived from the Simple Western Compass software and used to analyse data generated by WES. (**b**) An aligned dot plot with the length of cilia measured in each condition with ImageJ software segment tool. Student Welch’s *t*-test, *N* = 3 (>50 cells per *N*), ****, *p* < 0.0001, error bar represents ± SD. (**c**) Immunofluorescence images showing primary cilia stained with acetylated tubulin (green), pericentrin (red) and nucleus (blue) in WT, OCRL KO (A3 and A4) in PIP5K1A KD samples compared with their respective NT CTRL samples. Scale bar: 100 um.

In order to investigate whether our model is suitable for drug target screening in principle, we capitalised on the fact that downregulation of phosphatidylinositol-4-phospate 5-kinase alpha (PIP5 kinase α) has been shown to revert the F-actin phenotype and endocytosis defect in OCRL knockdown cells [15]. We speculated that downregulation of PIP5 kinase α might also rescue cilia length in OCRL KO cells. Using an siRNA approach, we successfully knocked down PIP5 kinase α in 3D proximal tubule cells. Subsequently, we measured cilia length and observed that knockdown of PIP5 kinase α could rescue the cilia length to wild-type levels in OCRL knockout clones. Interestingly, we also observed an effect of our siRNA on wild-type cells leading to a reduced cilia length, suggesting that a tight balance of PIP kinase and PIP phosphatase activity (Figure 5) regulates cilia length.

**Figure 6:**
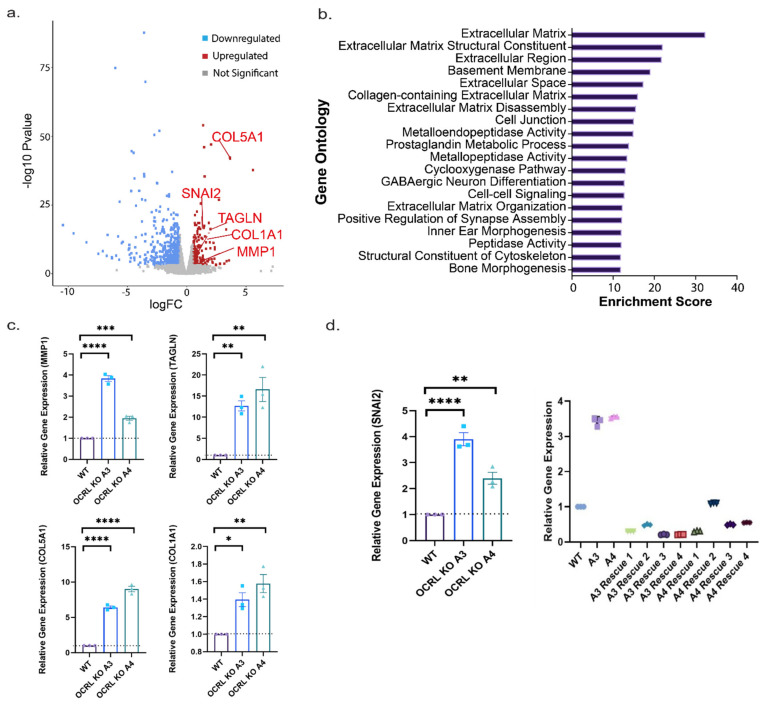
Differential gene expression: (**a**) volcano plot showing differentially expressed genes with a cut off of 0.5, and ECM- and EMT-related genes validated by qPCR are highlighted. (**b**) Gene Ontology (GO) terms filtered according to 161 differentially expressed genes. The highest score of 32.38, in blue, is the extracellular matrix in the cellular component. 17 (267)—17 genes on our list matched 267 known dysregulated genes in the extracellular matrix cellular component. (**c**) Other upregulated genes confirmed by qPCR. Relative gene expression was calculated by 2^−^^ΔΔ^^Ct^ with appropriate housekeeping genes. (**d**) SNAI2 expression was upregulated in the absence of OCRL, and the gene expression levels were reduced with the re-introduction of OCRL–GFP into the cells. Ordinary one-way ANOVA and Dunnett’s multiple comparison test, *N* = 3 (>50 cells per *N*). *, *p* < 0.05; **, *p* < 0.01; ***, *p* < 0.001; ****, *p* < 0.0001, error bar represents mean with ± SEM.

After demonstrating the suitability of our Lowe syndrome model for drug target validation, we also exploited our model to investigate the disease mechanism further. Using our in vitro model for Lowe syndrome/Dent II disease, we performed RNA-seq on tubules formed either from wild-type or OCRL KO cells under fluid flow. We found 161 genes to be differentially regulated in both OCRL KO (A3, A4) cell lines compared with wild-type HK-2 cells applying a cut off value of an adjusted *p*-value < 0.05 and a Log 2-fold difference in expression levels (Figure 6a). These 161 genes were further analysed by performing functional enrichment analysis. Gene ontology (GO) enrichment identified, in particular, genes that are implicated in EMT and extracellular matrix (ECM)-related mechanisms (Figure 6b). To validate our RNA-seq results, the upregulation of several genes of interest was confirmed by quantitative PCR. Specifically, we confirmed upregulation of SNAI2 and TAGLN, which are both known players in EMT, as well as COL1A1, COL5A1 (fibril forming collagens) and MMP1, which play an important role in fibrosis, a process which is characterised by elevated secretion and deposition of ECM proteins (Figure 6c). Importantly, we were able to show that expression levels of SNAI2 could be rescued after re-expression of OCRL (Figure 6d).

The upregulation of ECM genes, such as collagen in cells lacking OCRL expression, is of interest as interstitial fibrosis has been reported in Lowe syndrome. Thus, we investigated whether lack of OCRL leads not only to elevated collagen gene expression but also to increased collagen secretion and deposition.

**Figure 7:**
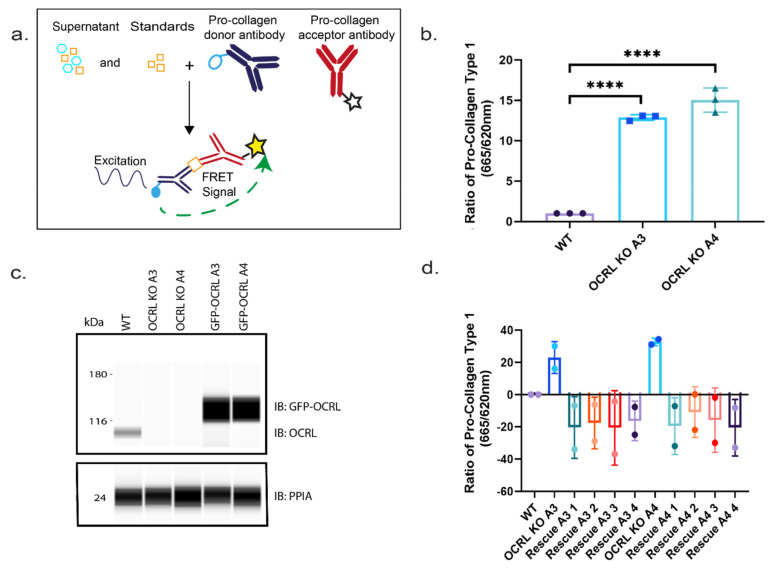
Collagen secretion: (**a**) the scheme of the FRET assay used to measure the levels of pro-collagen I in supernatants of cultured cells. (**b**) 3D experiment: graph showing quantitative analysis of collagen secretion measured via FRET assay in cultured cells’ supernatants. Ordinary one-way ANOVA and Dunnett’s multiple comparison test, *N* = 3 (>50 cells per *N*), ****, *p* < 0.0001, error bars represent mean with ± SD. (**c**) Western blot showing the expression of GFP-tagged OCRL at approximately 135 kDa in OCRL KO clones with respect to HK-2 WT cell, with the expression of OCRL at approximately 110 kDa. (**d**) Graph showing the quantitative analysis of collagen secretion measured via FRET assay in cultured cells’ supernatants. Rescue cell lines showing decreased collagen secretion with respect to WT and a scatter dot plot showing the mean with SD, *N* = 2.

Using a fluorescence resonance transfer (FRET)-based assay, we could demonstrate that the amount of pro-collagen I secreted into the medium was elevated for both OCRL KO cell lines compared to wild-type HK-2 cells. To further confirm that this effect results from lack of OCRL expression, we generated stable cell lines from our OCRL KO clones A3 and A4 re-expressing OCRL. Importantly, the increase in elevated collagen secretion reverted even below wild-type collagen secretion levels after re-expression of OCRL. The fact that secretion was reduced below wild-type levels was likely due to the strong overexpression of OCRL compared to endogenous OCRL expression levels (Figure 7).

**Figure 8:**
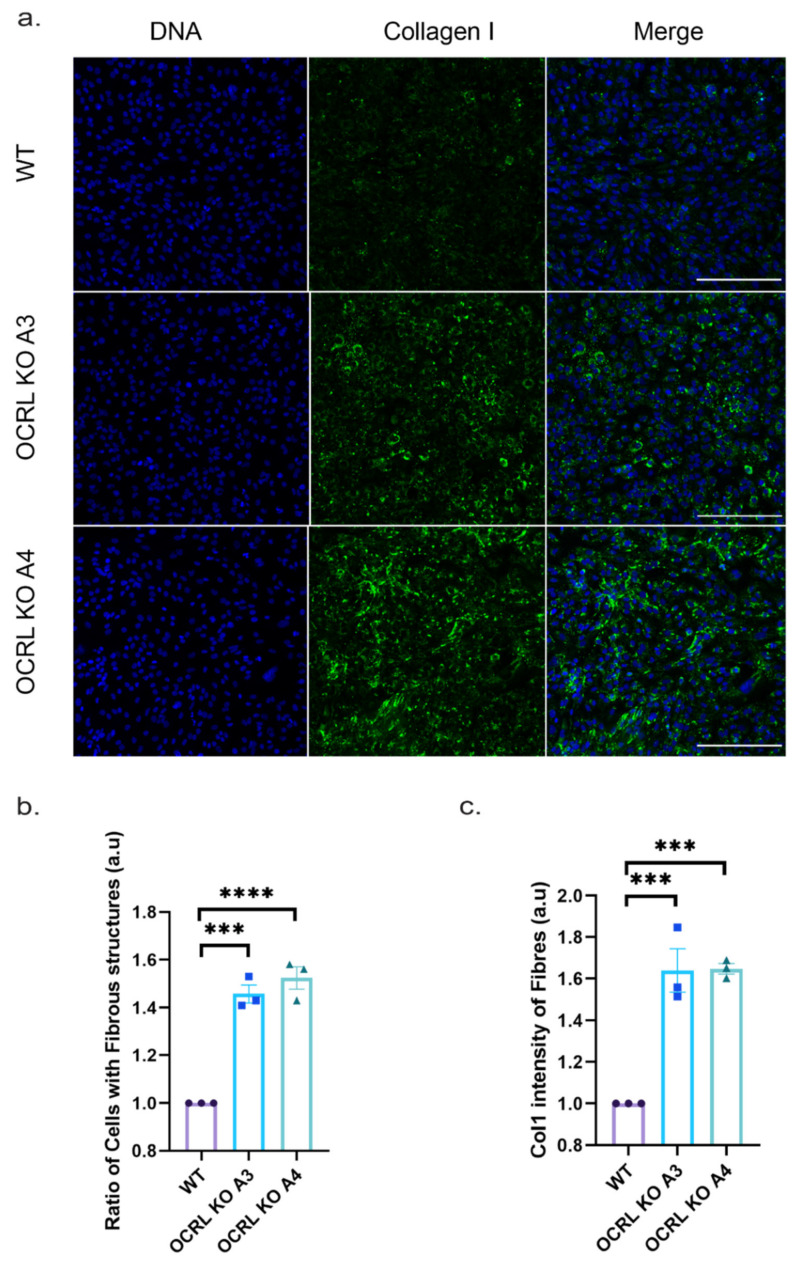
Collagen deposition: (**a**) 2D experiment: Immunofluorescence images captured by an InCell 6000 when cells were cultured for 7 days and stained with Col 1 antibody without permeabilising the cells to stain for ECM deposition (green). Scale bar: 5 µm. (**b**) 2D experiment: Graph showing increased ratio of cells with fibrous structures in OCRL KO cells when stained for collagen. (**c**) 2D experiment: graph showing increased collagen fibres deposited on OCRL KO cells measured by normalising the collagen staining intensity by the number of cells. Ordinary one-way ANOVA and Dunnett’s multiple comparison test, *N* = 3 (>50 cells per N), ***, *p* < 0.001; ****, *p* < 0.0001, error bar represents the mean with ± SEM.

Finally, we also tested whether the increased pro-collagen I (COL1A1) expression levels lead to an increase in collagen fibre deposition. Applying a custom-made algorithm (see Section 4) to identify collagen fibres using collagen immunofluorescence, we quantified the ratio of cells displaying collagen fibres and quantified their overall intensity. Both OCRL KO clones showed significantly more cells with collagen fibres and an overall increase in collagen fibre intensity (Figure 8). Thus, we concluded that lack of OCRL expression leads to increased pro-collagen I mRNA expression levels, increased collagen secretion and increased collagen fibre formation, potentially contributing to interstitial fibrosis, a phenotype observed in animal model systems for Lowe syndrome and Lowe syndrome patients.

## 3. Discussion

The major cause for decreased life expectancy in Lowe syndrome is the development of chronic kidney disease and associated complications [38]. Thus, understanding the renal phenotype in Lowe syndrome is of major importance and the availability of suitable model systems to study these diseases is critical.

Here, we presented a novel in vitro model for the renal phenotype of Lowe syndrome using organ on chip technology. Importantly, our model is fully amenable for drug target identification and validation and is suitable to investigate underpinning molecular disease mechanisms.

Using CRISPR/Cas9 technology, we generated proximal tubule HK-2 cell lines lacking OCRL expression. In line with previous reports, such OCRL KO cell lines recapitulate important phenotypes of Lowe syndrome including reduced megalin-dependent endocytosis and increased actin polymerisation around endosomes [15,16,17,34].

We have implemented these cell lines into a microfluidic platform (OrganoPlate) that allowed for the growth of 3D epithelial tubules together with the application of fluid flow shear stress [35,36]. It has become clear that 3D architecture and mechanical forces, such as fluid flow stress, are important regulators of cellular homeostasis and both are lacking from standard static 2D cultures [39,40]. We demonstrated, here, that under fluid flow, OCRL KO HK-2 cells have shorter cilia compared to wild-type HK-2 cells. This is in line with previous reports of impaired ciliogenesis in HK-2 cells lacking OCRL and reduced cilia length in dermal fibroblasts isolated from Lowe syndrome patients cultured under static conditions [27,41].

Using cilia length as a readout for the Lowe syndrome phenotype, we demonstrated that our Lowe syndrome in vitro model can be used for drug target identification, as we were able to rescue cilia length using an siRNA approach targeting PIP5 kinase α. Previously, it was shown that knocking down PIP5 kinase α can restore endocytosis and endosomal F-actin in OCRL knockdown HK-2 cells or ameliorate the pronephric endocytosis defect in zebrafish [15,17]. Recently, it was also demonstrated that targeting phosphatidylinositol-3-kinase (PI3kinase) using a small molecule inhibitor approach can improve proximal tubule function in vitro and in a mouse model of Lowe syndrome and Dent II disease [34]. Thus, our human in vitro model could also prove useful in small molecule screens or drug validation, in particular if further improved by replacing HK-2 cells by proximal tubule cells isolated from patients or by generating proximal tubule cells from induced pluripotent stem cells [41,42]. We demonstrated, recently, the on-chip differentiation of pluripotent stem cells into intestinal tubules [37].

We also exploited our Lowe syndrome model to further investigate the underpinning disease mechanisms. The RNA-sequencing experiments performed on 3D tubules grown in the presence of fluid flow stress revealed significant differences in gene expression between wild-type and OCRL KO HK-2 cells. Among other proteins, we observed the upregulation of the transcription factor SNAI2 (also known as Slug) in HK-2 cells lacking OCRL compared to wild-type OCRL. Importantly, this upregulation was specific to the OCRL knockout as we could rescue SNAI2 expression levels by re-expressing OCRL in OCRL KO cells. SNAI2 is a transcription factor playing a major role in development, but it is also known as a prototypical EMT transcription factor [43]. EMT is the process of epithelial cells acquiring mesenchymal features. This process plays an important role during development but also in disease initiation and progression in cancer and fibrosis. In the context of kidney fibrosis EMT had initially been proposed to be the main process to generate interstitial myofibroblasts, but lineage tracing experiments confirmed that only approximately 5% of myofibroblasts trace their origin back to epithelial cells [44]. However, inhibiting EMT by knocking out EMT transcription factors SNAI1 or Twist selectively in proximal tubule cells reduced ECM deposition and ameliorated interstitial fibrosis in mouse models of kidney fibrosis [45,46]. Interestingly, in recent years, our view of EMT has changed dramatically, instead of promoting a fate of either being epithelial or mesenchymal, it has become clear that EMT reflects rather a continuum of mixed phenotypes where epithelial cells acquire different degrees of mesenchymal properties [47]. Thus, proximal tubule cells undergoing EMT usually do not convert into fibroblasts but remain an integral part of the tubule acquiring only a partial EMT status [44]. This partial EMT status is associated with changes in the secretome and proliferation rate and promotes inflammation and fibrosis [45,46].

Our RNA-seq experiments are in line with the idea that HK-2 cells lacking OCRL undergo partial EMT. We confirmed upregulation of SNAI2 but also of SNAI2 downstream targets such as MMP1, COL1A1 and COL5A1 [44,48]. Moreover, we also showed that HK-2 OCRL KO cells display increased COL1A1 secretion leading to increased extracellular collagen fibre deposition, which may contribute to interstitial fibrosis as observed in Lowe syndrome and Dent II disease patients.

Currently, it is unclear why SNAI2 is upregulated in HK-2 OCRL KO cells; however, we also observed upregulation of TAGLN and overexpression of TAGLN can promote EMT in part via upregulation of SNAI2 [49]. TAGLN is a target of TGF-β signalling and its upregulation could reflect altered TGF-β signalling in HK-2 OCRL KO cells [50]. In summary, our data suggest that upregulation of SNAI2 and possibly partial EMT could contribute to the progressive kidney phenotype observed in Lowe syndrome and Dent II disease; however, confirmation of these results in animal model systems and Lowe syndrome/Dent II disease patients is necessary.

## 4. Materials and Methods

### 4.1. Antibodies and Reagents

The following antibodies were used in this study: Anti-OCRL (HPA012495, 1:500 for IB and IF, Atlas Antibodies, Bromma, Sweden), N-cadherin (sc-59987, 1:100 for IB, Santa Cruz Biotechnology, Inc., Dallas, TX, USA), AQP1 (B-11) (sc-25287, 1:500 for IB, Santa Cruz Biotechnology, Inc., Dallas, TX, USA), Na^+^/K^+^-ATPases (H-3) (sc-48345, 1:1000 for IB, Santa Cruz Biotechnology, Inc., Dallas, TX, USA), EEA1 (610456, 1:500 for IF, BD Biosciences, San Jose, CA, USA), acetylated tubulin (T6793, 1:2000 for IF, Sigma-Aldrich, St. Louis, MO, USA), PCNT (ab4448, 1:1000 for IF, Abcam, Cambridge, MA, USA), CI-M6PR 2G11 (ab2733, 1:100, Abcam, Cambridge, MA, USA) along with EEA1 (C45B10, 1:100 for IF), Alexa Fluor™ 488 Phalloidin (A12379, 1:4000 for IF, Thermo Fisher Scientific, Waltham, MA, USA), Anti-GST (27457701, 1:1600 for IF, GE Healthcare, Chicago, IL, USA), collagen I (ab34710, 1:500 for IF, Abcam, Cambridge, MA, USA), PPP1R1A (ab40877, 1:100 for capillary IB, Abcam, Cambridge, MA, USA), PIP5K1α (9693S, 1:100 for capillary IB, Cell Signaling Technology, Danvers, MA, USA) and TBP (8515S, 1:500 for capillary IB, Cell Signaling Technology, Danvers, MA, USA).

Reagents used were TGF-β1 protein (240-B-002, R&D Systems, Minneapolis, MN, USA), 20 µg/mL, SuperScript™ II Reverse Transcriptase (18064014, Thermo Fisher Scientific, Waltham, MA, USA), Phusion High-Fidelity DNA Polymerase (F530S, Thermo Fisher Scientific, Waltham, MA, USA), Restriction Enzymes HindIII (FD0504), KpnI (FD0524), BamHI (FD0055) and NotI (FD0593) were from Thermo Fisher Scientific (Waltham, MA, USA), Collagen-I (5 mg/mL) (3447-020-01, AMSBIO, Cambridge, MA, USA), 1 M HEPES (15630-122, Thermo Fisher Scientific, Waltham, MA, USA), NaHCO_3_, pH 9.5 (Sigma, S5761), TRITC Dextran 155 kDa (Sigma, T1287), FITC Dextran 20 kDa (FD20S, Sigma-Aldrich, St. Louis, MO, USA), M-PER™ Mammalian Protein Extraction Reagent (78501, Thermo Fisher Scientific, Waltham, MA, USA) and Halt Protease and Phosphatase Inhibitor Complex (78428, Thermo Fisher Scientific, Waltham, MA, USA), ON-TARGETplus OCRL siRNA (L-010026-00-0005, Dharmacon, Inc., Lafayette, CO, USA), ON-TARGETplus PIP5K1A Human siRNA (L-004780-00-0005, Dharmacon, Inc., Lafayette, CO, USA) and ON-TARGETplus Non-targeting pool (D-001810-10-05, Dharmacon, Inc., Lafayette, CO, USA).

### 4.2. Cell Culture, Gene Editing and Transfection

HK-2 cells were obtained commercially from ATCC. Cells were cultured and maintained at 37 °C with 5% CO_2_ in DMEM:F12 HAMs basal medium (11039-021, Thermo Fisher Scientific, Waltham, MA, USA) supplemented with 1% of ITS (I1884, Sigma-Aldrich, St. Louis, MO, USA), 36 ng/mL of hydrocortisone (H0135, Sigma-Aldrich, St. Louis, MO, USA), 10 ng/mL of hEGF (E9644, Sigma-Aldrich, St. Louis, MO, USA), 40 pg/mL of 3-iodothyronine (T5516, Sigma-Aldrich, St. Louis, MO, USA), 10% of Foetal bovine serum (16140-071, Thermo Fisher Scientific, Waltham, MA, USA) and 1% of pen/strep (P4333, Sigma-Aldrich, St. Louis, MO, USA). Cells were passaged 1:4 ratio once 80% confluent and medium were refreshed every 2–3 days.

Guide RNA sequence ATAATCCAGTTGCATGAGA (target sequence) AGG (PAM) from Exon 3 was selected using CRISPOR software. The gRNA sequence sub-cloned in the parent vector pSpCas9(BB)-2A-GFP (PX458) was ordered from GenScript^®^ (Piscataway NJ, USA). Transfection of the plasmid was performed using Lipofectamine™ 2000 (11668027, Thermo Fisher Scientific, Waltham, MA, USA) when the cells were 80% confluent. The cells were starved by leaving the DNA:lipid mixture in reduced serum conditions using Opti-MEM (11058-021, Thermo Fisher Scientific, Waltham, MA, USA) for 4 h to increase efficiency. After 48 h, cells were collected and GFP-positive single cells were sorted in 96-well plates using BD FACS Aria II (Flow Cytometry Core Facility, The University of Sheffield, Sheffield, UK). Colonies were formed from single cells by maintaining the culture at 37 °C with 5% CO_2_ for 2–3 weeks. These were sequentially expanded, and protein was extracted from these cells to perform Western blot analysis. Clones with OCRL KO were then expanded further to use for future experiments. Genomic DNA was extracted from transfected pool of cells using the Blood & Cell Culture DNA Mini Kit (13323, QIAGEN Inc., Germantown, MD, USA) following the manufacturer’s instructions, the region of interest was amplified and PCR products were sent for sequencing to determine the position of the in-del and stop codon formation leading to truncation of protein expression.

### 4.3. T7E1 Assay

Genomic DNA was extracted from transfected pool of cells as mentioned earlier, and the target gene region in OCRL (Exon 3) was amplified using OCRL specific primers (Forward Primer: 5′-CACCACTAGCATCCTTTTAGGC-3′ and Reverse Primer: 5′-AGAGAAAGTATCATCTCCTCAATGT-3′). T7 Endonuclease I (M0302S, New England Biolabs, Hitchin, UK) was used to digest PCR products, which were denatured and annealed to facilitate the formation of heteroduplex DNA. The enzyme was incubated with PCR product for 90 min at 37 °C. The reaction was stopped by adding 0.25 M EDTA and the digested products were separated using 2% agarose gel electrophoresis to check for fragmented DNA.

### 4.4. GST–RAP Protein Production and Purification

The cDNA was reverse transcribed from the total RNA extracted from HK-2 wild-type cells. The RAP cDNA was amplified using primers flanked by BamHI and XhoI restriction sites (Forward primer 5′-GATCGGATCCGGGATGATGGCGCCGCGGAGGGTCA and Reverse Primer: 3′-GATCCTCGAGTCAGAGTTCGTTGTGCCGAGCTC). The PCR product was sub-cloned into PGEX-6P-1 vector, which harbours a GST tag sequence before its multiple cloning site. The cloned plasmid was transformed into BL21(DE3) Competent Cells (71397-3, Novagen Inc., Madison, WI, USA) and plated on ampicillin resistant agar plates. A colony was cultured, and protein expression was induced using 1 mM IPTG. Bacteria were harvested and sonicated to release proteins. Supernatant was incubated with equilibrated GST beads overnight at 4 °C. The GST tagged RAP protein was eluted and purified using Slide-A-Lyzer™ Dialysis Cassettes (66005, Thermo Fisher Scientific, Waltham, MA, USA ) with 1X PBS buffer exchange.

### 4.5. Immunofluorescence Analysis

Co-localisation Assay: Co-localisation analysis was performed on Z stack images obtained by Perkin Elmer Spinning disc microscopy at 60X magnification using the ImageJ software plug-in Jacop (Just another co-localization plug-in). Images were thresholded, and the Pearson coefficient and Mander’s pixel fraction overlapping co-efficient were calculated after reducing the background signal by setting-up a threshold. F-Actin quantification: F-Actin staining intensity was measured in Z stack images acquired by Perkin Elmer Spinning disc microscopy at 60X magnification using ImageJ software.

GST–RAP Uptake Assay: Appropriate amount of GST-RAP protein was incubated diluted in DMEM:F12 basal medium for different time points. The cells were washed with 1X PBS 4 times before proceeding with fixation with 4% PFA and staining to detect GST–RAP. The images were acquired using the water immersion objective of a confocal microscope (Perkin Elmer Operetta CLS™, PerkinElmer, Inc., Waltham, MA, USA). The analysis sequence to quantify GST–RAP was created using Harmony 4.8.2127.198 software. The imaging protocol 96-well assay plate was set to image 5 fields and two z-planes with a 2 µm separation. Input images were corrected with a basic flat field correction and analysed on individual planes. Nuclei were identified from the Hoechst 33,342 staining to identify the number of cells and to serve as a region of interest (ROI) for the cytoplasm detection. The cytoplasm of the cells was then identified by local intensity thresholding of the Alexa 488 signal. The RAP-positive spots were detected within the entire cell area through a spot detection step. The mean and sum intensity of the spots were calculated as well as their area and roundness. The intensities obtained in an image were normalised vs. number of cells. Mean normalised intensity values across samples were taken into consideration to quantify the GST–RAP uptake in wild-type HK-2 cells vs. OCRL KO cells.

### 4.6. GST–RAP Ligand Binding Kinetics

GST–RAP ligand was incubated with cells at different concentrations ranging from 0.5–8 µg/mL (2-fold increase) for 1 h on ice to avoid ligand internalisation by endocytosis. Cells were washed thrice with basal medium to remove any unbound ligands. Cells were then incubated with GST antibody conjugated with Alexa-fluor at 488 nm for 1 h (1:100 dilution in FACS staining buffer). Cells were then washed thrice with FACS staining buffer to remove unbound primary antibody. Cells were analysed with a flow cytometer and 10,000 events were recorded per sample. Median intensity value from no-ligand control was subtracted from all other samples to remove background signal. The data were analysed using GraphPad Prism’s nonlinear regression analysis. Binding saturation with one site-specific binding model was chosen to derive the Bmax and kd values.

### 4.7. Homogenous Time Resolved Fluorescence (HTRF) Assay

The HTRF assay kit (63ADK014PEG, Cisbio, Bedford, MA, USA) was used to measure the amounts of secreted pro-collagen I in the supernatant of cultured cells using the manufacturer’s instructions. Briefly, 16 µL of supernatants and standard samples were added to the plate in technical triplicates. Four microliters of diluted Cryptate/d2 antibody solution were added per well, and the HTRF plate was centrifuged at 1000 RPM for 1 min. The samples were incubated for 3 hours at RT while covered with a black lid or aluminium foil to protect from light. The fluorescence of the acceptor molecule was measured using a plate reader at 665 nm wavelength.

### 4.8. TGF-β1 Trigger and Collagen Deposition Assay

The TGF-β1 protein was incubated with cells at 10 ng/mL and ALK5-inhibitor compound was used as a negative control in this assay in a dose–response manner (0.001–10 µM). Cells stained for collagen deposition were imaged using an Incell 6000 microscope and collagen deposition was measured using an algorithm with three criteria. Segmentation criteria were set to identify and de-cluster the nucleus such that all cells were included in the DAPI channel except for ones with uneven and unhealthy-looking nuclei. The FITC channel with collagen staining was set-up to detect fibre-like structures, and all the background unspecific noise was cut-off by setting the intensity threshold value as fibrous-like collagen structures were accumulating and cells were migrating towards each other. The ratio of cells with a mask created around the cell (cell body) with collagen fibre-like structures were calculated in each condition vs. cells without the collagen fibrous structures.

### 4.9. Proximal Tubule on a Chip Culture

A 3-lane OrganoPlate from Mimetas B.V. (Catalogue Number: 4004-400-B) was used to culture 3D proximal tubule cultures with HK-2 cells. The middle channel was filled with 2 µL of collagen I, 4 mg/mL after mixing collagen I (5 mg/mL) with 1 M HEPES, pH 7.2–7.5 and NaHCO_3_, pH 9.5 in 8:1:1 ratio on ice. The plate was incubated at 37 °C with 5% CO_2_ in the incubator for 15 min to polymerise the ECM and solidify. The upper channel was filled with 2 µL of cells such that each chip had 15,000 cells. The plate was kept standing at a 75° angle for 1 h to let all the cells adhere against the ECM. The upper channel outlet was filled with 50 µL of culture medium and the plate was placed on a rocking platform. The platform was set to rock and change direction every 8 min at 7° angle. The medium was changed every 2–3 days and tubule formation was complete by day 4 or 5.

### 4.10. Barrier Integrity Assay

All culture medium was removed from the upper inlet and outlet of the OrganoPlate. Twenty-five microlitres of medium were added in all inlets and outlets. The plate was kept on a rocker for 5 min. TRITC 155 kDa and FITC 20 kDa dextran were diluted 1:50 using culture medium. Twenty microlitres of medium were added in all middle channels and lower channels inlets and outlets. Forty and thirty microlitres of dextran working solution were added to the upper channel inlet and outlet, respectively. The plate was imaged by molecular devices confocal microscope using TRITC and FITC channels. The microscope was set to take one image of all the chips every 2 min for a time period of 14 min. ImageJ was used to measure the fluorescence intensity of the top channels vs. the gel channels over the 14 min time period. The ratio of the signal intensities determined the leak tightness of the tubules.

### 4.11. Immunofluorescence in OrganoPlate

Medium was removed from all wells in the OrganoPlate. Fifty microlitres of 4% PFA were added in the upper channel inlets and outlets to create a gradient such that the PFA flowed through the lumen of the tubule over an incubation time of 20 min at room temperature. The PFA was aspirated from all the wells and the plate was washed by adding 1X PBS like above and incubated for 5 min. This step was repeated 3 times to get rid of all PFA. The cells were permeabilised by adding the permeabilisation buffer (0.3% Triton X-100 in 1X PBS) incubated for 10 min at room temperature. The permeabilisation buffer was removed and washed with washing solution (4% FBS in 1X PBS) for 5 min. Blocking buffer (2% FBS, 2% BSA, 0.1% Tween20 in 1X PBS) was added and incubated at room temperature for 1 h. Blocking buffer was removed and desired primary antibody was diluted in blocking buffer and added in the channels. Forty microlitres in the upper channel inlet, 30 µL in the upper channel outlet, 20 µL in the lower channel inlet and outlet and incubated at 4 °C overnight. Primary antibody was removed, and channels were washed with washing solution 3 times for 5 min each. Secondary antibody against the primary antibody was diluted 1:500 in blocking buffer and was added in the channels and incubated for 1 h at room temperature. Buffer with secondary antibody was removed and cells were washed with washing solution thrice for 5 min each. Channels and cells were washed with 1X PBS once plate was covered in aluminium foil to protect from light.

### 4.12. RNA Sequencing and Data Analysis

Three biological replicates of 3D HK-2 cells (wild type and OCRL KO) were cultured in OrganoPlate for 4 days with fluid flow before RNA was isolated and purified from the OrganoPlate using the RNeasy Micro Kit (74004, QIAGEN Inc., Germantown, MD, USA). Culture medium was aspirated from the channels and discarded. Forty microlitres and 30 µL of lysis buffer from the kit were added to the upper channel inlet and outlet, respectively and incubated with the cells for 5 min. Lysate from the cells were collected (70 µL in total), 5 chips were pooled in and 350 µL total lysate was purified using the columns from the kit following manufacturer’s instructions. Genomic DNA was removed using DNASE I treatment which is part of the kit. Purified RNA’s concentration and purity was measured using Nanodrop and 5200 Fragment Analyzer (M5310AA, Agilent, Santa Clara, CA, USA) following the manufacturer’s instructions.

To perform standard RNA-seq, 10 µL of RNA with minimum concentration of 50 ng/µL was sent to GENEWIZ, LLC. (South Plainfield, NJ, USA), which performed RNA library preparation with polyA selection for sequencing with Illumina HiSeq, 2 × 150 bp configuration, single index, per lane. The ~350 M raw paired-end reads per lane per sample was provided after sequencing, and the bio-informatics team at Galapagos BV performed the data analysis. Briefly, raw data were pre-processed using a workflow consisting of trimming reads to remove adapters, indexes and bad-quality bases using Trimmomatric [51] and quality control on trimmed reads using FastQC. Filtered reads were aligned/mapped to the reference genome build using STAR [52] and pseudo-mapped against the reference transcriptome using Kallisto [53]. The mapping quality was assessed focusing on coverage of splice junctions; rates of splicing in known or novel junctions; read coverage of splice junctions; gene body coverage and potential 5′-3′ bias; insertions–deletions: the rate of occurrence and the lengths using QoRTS [54]. The estimated counts from the Kallisto abundance estimations (at the transcript level) were summarized at the gene-level by using the R package txtimport [55] using Ensembl annotations and DESeq2 (R/Bioconductor package) is used to fit a negative binomial model to the data [56]. Results tables with the main outputs the log2-fold change (FC), *p*-values and adjusted *p*-values (false discovery rate–FDR) were generated from which the volcano plot and gene ontology analysis were performed.

### 4.13. RNA Interference, Reverse Transcription and Quantitative PCR (qPCR)

The siRNA transfection in OrganoPlate was performed by diluting the siRNA’s in Opti-MEM to a final optimised concentration. Lipofectamine RNAiMAX Transfection Reagent was diluted 1:50 with Opti-MEM. The siRNA:lipid were mixed in a 1:1 ratio and left to incubate at RT for 15–20 min to form RNA:lipid complexes. During the above incubation, medium from the plates were removed and 50 µL was added in the upper channel inlet and outlet. Ten microlitres of siRNA/RNAiMAX complexes were added to the upper channel inlet and outlet. Plates were then incubated at 37 °C, 5% CO_2_ on a rocker set at 7° angle to change every 8 min for 48 h before cells were harvested for RNA/protein isolation for further analysis as described earlier.

The RNA was used to reverse transcribe to get cDNA using TaqMan Reverse Transcription Kit (N8080234, Applied BioScience, Foster City, CA, USA). The cDNA was serially diluted 2× times and amplification efficiency of both target gene and housekeeping gene using Taqman probes was compared to choose the right housekeeping gene to normalise for relative quantification of gene expression levels. The qPCR was performed using Light Cycler 480™ (Roche, Basel, Switzerland), Ct values obtained for both target gene (FAM dye) and housekeeping gene (VIC dye) were used to calculate relative gene expression levels using Livak method (2^−ΔΔCt^ method).

List of Assay IDs used for Taqman probes: SNAI2-Hs00161904_m1; COL1A1-Hs00164004_m1; COL5A1-Hs00609088_m1; TAGLN-Hs00162558_m1 and MMP1-Hs00899658_m1.

### 4.14. Statistics and Data Analysis

All statistical analyses were performed using GraphPad Prism software. Statistical significance of the data obtained from multiple independent experiments were tested using the *t*-test for two data sets or ordinary one-way ANOVA and Dunnett’s multiple comparison test for more than two groups unless otherwise specified. Co-localisation analysis was performed using Mander’s overlap co-efficient using the Jacop plug-in from ImageJ software.

## Data Availability

The data sets generated during and/or analysed during the current study are available in the GEO repository (GSE171848).

## Supplementary Materials

The following are available online at https://www.mdpi.com/article/10.3390/ijms22105361/s1, Figure S1: Confirmation of OCRL KO stable cell lines; Figure S2: RAP–GST Binding to megalin assay; Figure S3: Total Megalin receptors at plasma membrane; Figure S4: F-Actin staining on endosomes, Figure S5: Transport defect from endosomes to TGN.
