# Supplementary material for: A 3D Renal Proximal Tubule on Chip Model Phenocopies Lowe Syndrome and Dent II Disease Tubulopathy
Source: Int J Mol Sci. 2021 May 19;22(10):5361. doi: 10.3390/ijms22105361 (PMC8161077; doi:10.3390/ijms22105361)
Supplement: Supplementary file 1 [file ijms-22-05361-s001.zip › ijms-1221436-supplementary.pdf]

International Journal of *Molecular Sciences*

MDPI

Supplementary figures

a. WT OCRL Exon 3

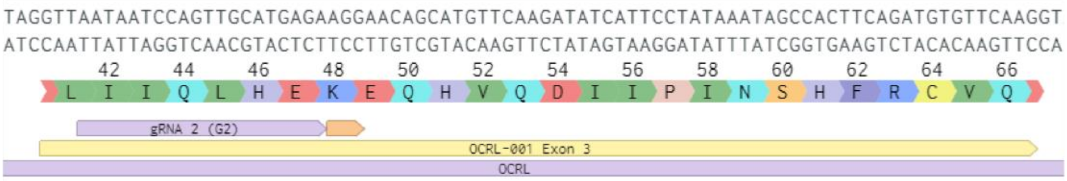

b. OCRL KO Clone A3 (1bp deletion - 58aa)

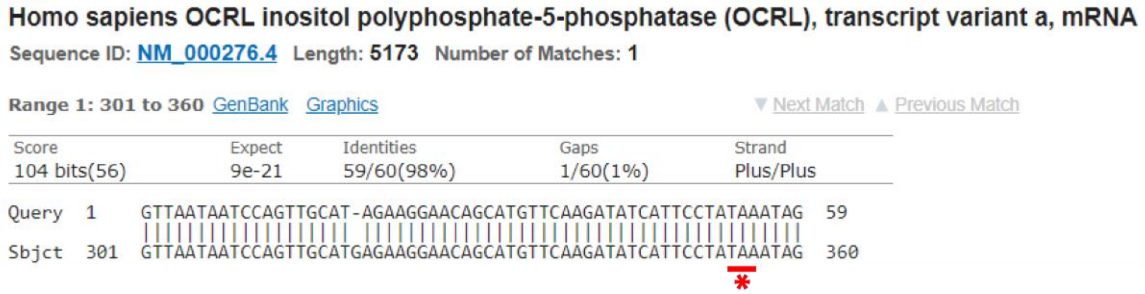

c. OCRL KO Clone A4 (1bp insertion - 60aa)

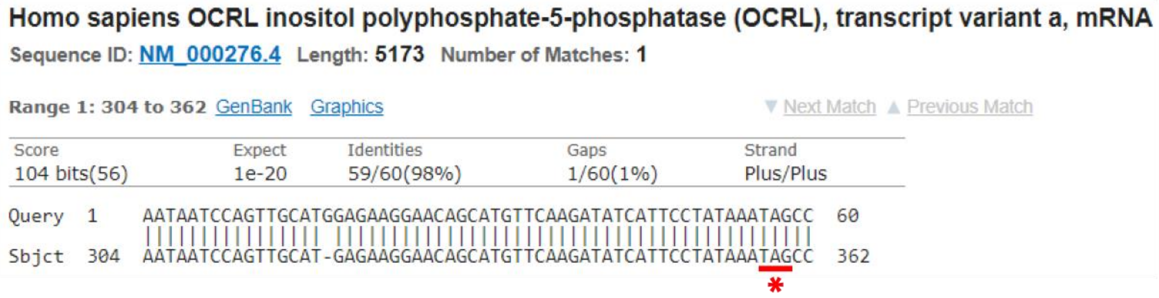

Figure S1: Confirmation of OCRL KO Stable Cell lines: (**a**). Schematic representation of the target region of G2 gRNA on Exon 3 of OCRL gene. (**b & c**). Sequencing results confirming the indels leading to the stop codon in both KO clones.

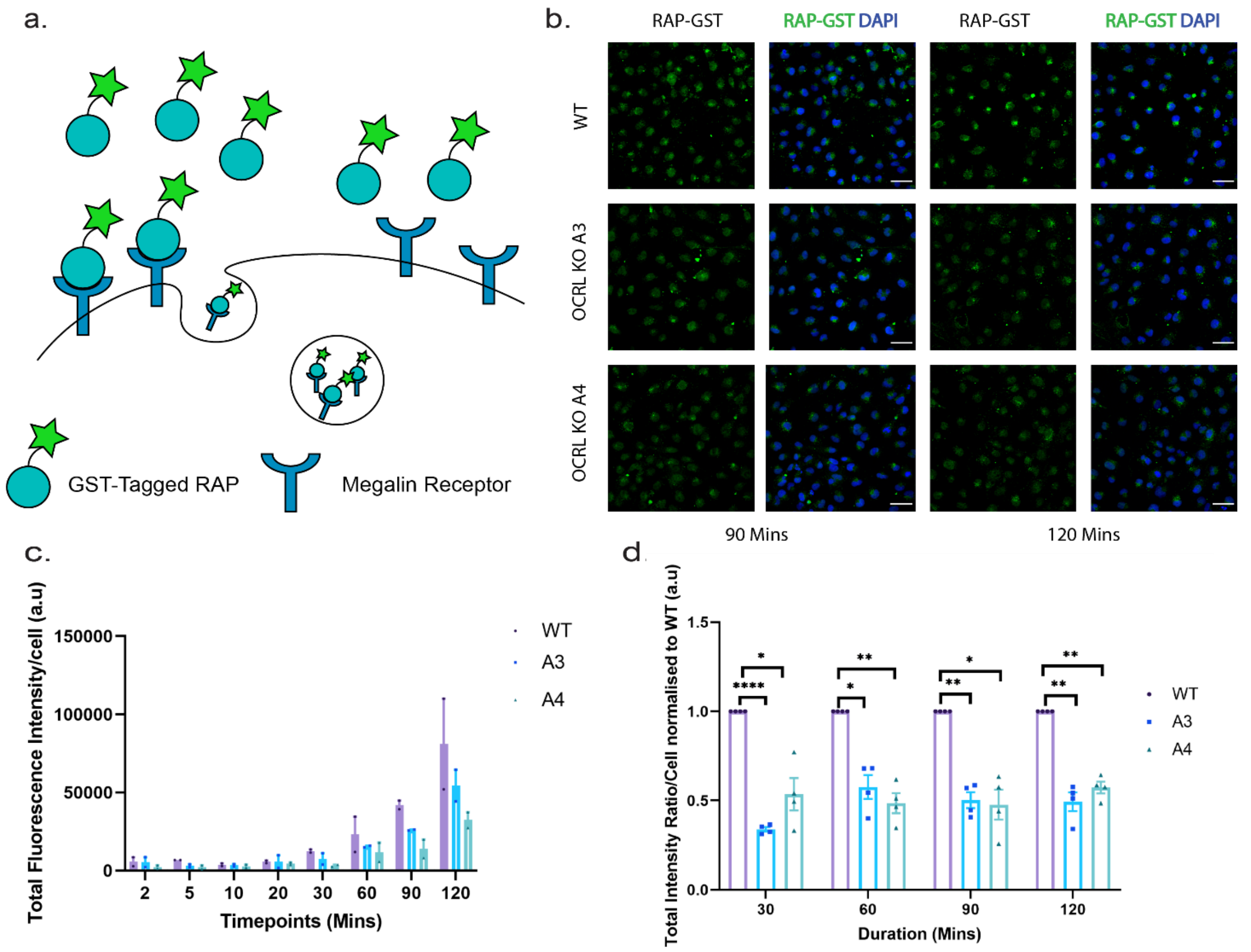

Figure S2. RAP-GST Binding to Megalin Assay: (**a**) Graphical representation of the experimental set up. GST-Tagged RAP protein was purified and provided to the cells to bind with Megalin receptors. (**b**) Immunofluorescence analysis of internalisation of RAP-GST by Megalin receptor in WT and OCRL-KO HK-2 clones. The cells were incubated with 2.5 µg/mL of RAP-GST for 120 min and 90 min in serum-free medium at 37 °C, images were taken with confocal microscope (Operetta CLS™ by Perkin Elmer). Scale Bar: 50µm. (**c**) Increased uptake efficiency with different time-points in WT, OCRL KO (A3 and A4). Graph shows values from 2 min and increasing up to 120 min, $N = 2$, error bar represents mean with ± s.e.m. (**d**) Graph showing 2-fold increase in the uptake efficiency when intensity values were normalised vs WT. Two-way ANOVA with Geisser-greenhouse correction and Dunnett's multiple comparison test, $N = 4$, *, $p < 0.05$; **, $p < 0.01$; ***, $p < 0.001$; ****, $p < 0.0001$, error bar represents mean with ± s.e.m.

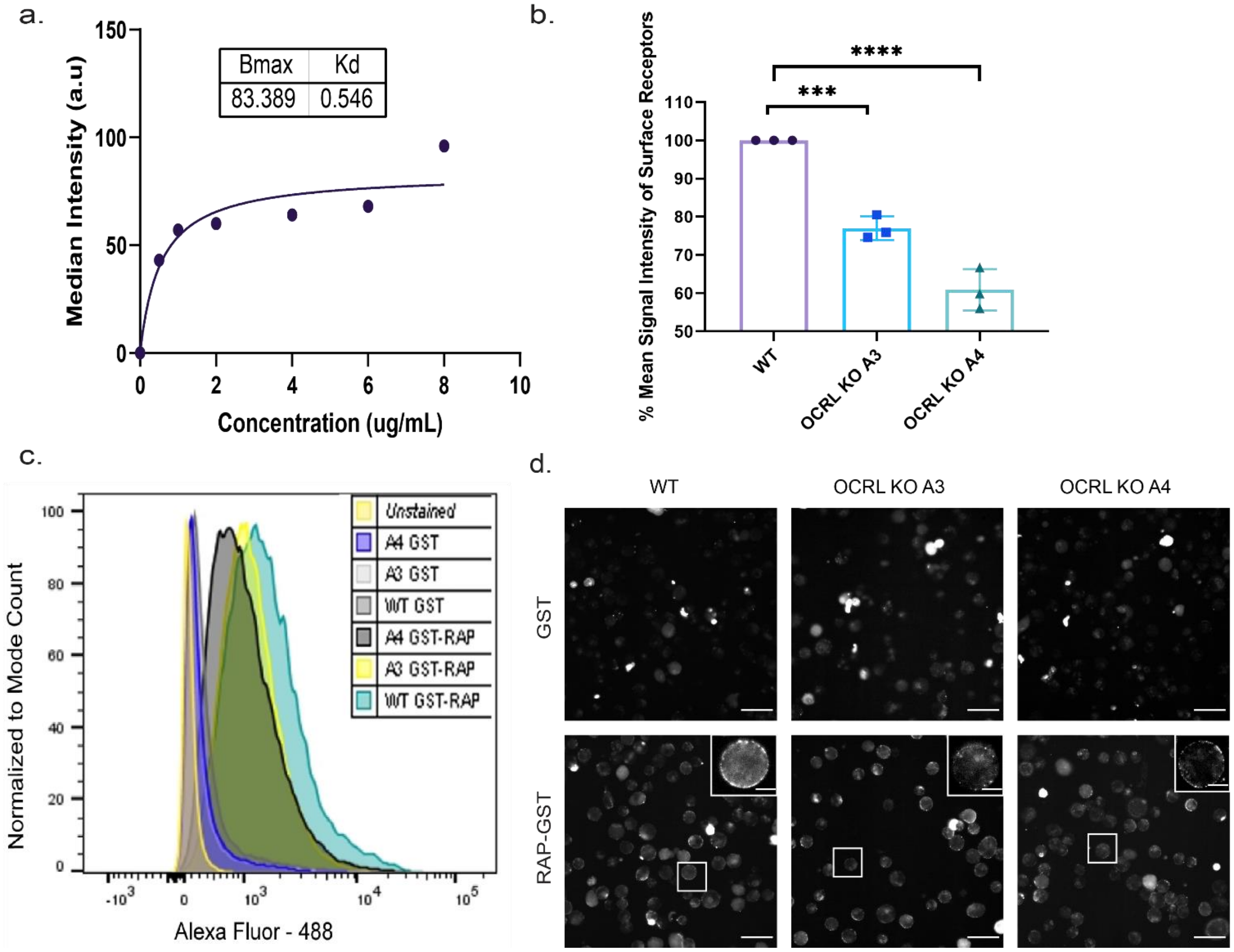

Figure S3. Total Megalin receptors at plasma membrane: (**a**) Dynamic range of GST-RAP ligand binding kinetics calculating K(max) using median fluorescence was obtained via FACS experiment. Each point represents a 2-fold increase of GST-RAP ligand concentration provided to the cells. (**b**) % of Megalin surface receptors in OCRL KO cells normalised to WT, based on fluorescence intensity values from FACS experiment. Ordinary one-way ANOVA and Dunnett's multiple comparison test, $N = 3$, *, $p < 0.05$; **, $p < 0.01$; ***, $p < 0.001$; ****, $p < 0.0001$, error bar represents mean with ± s.e.m. (**c**) Graph showing fluorescence signal measured using 10,000 cells from each condition, the shift in fluorescence values from WT vs OCRL KO cells shows the receptor availability at plasma membrane. (**d**) Images of WT and OCRL KO (A3 and A4) cells in suspension after staining and before running through a flow cytometer. Image shows staining signal at the plasma membrane of the cells, there is visibly more staining in the cells in WT when compared with cells in A3 and A4. Images were acquired with confocal microscope (In Cell Analyzer 6000 from GE Healthcare) Scale Bar: 50µm (Inset: 10µm).

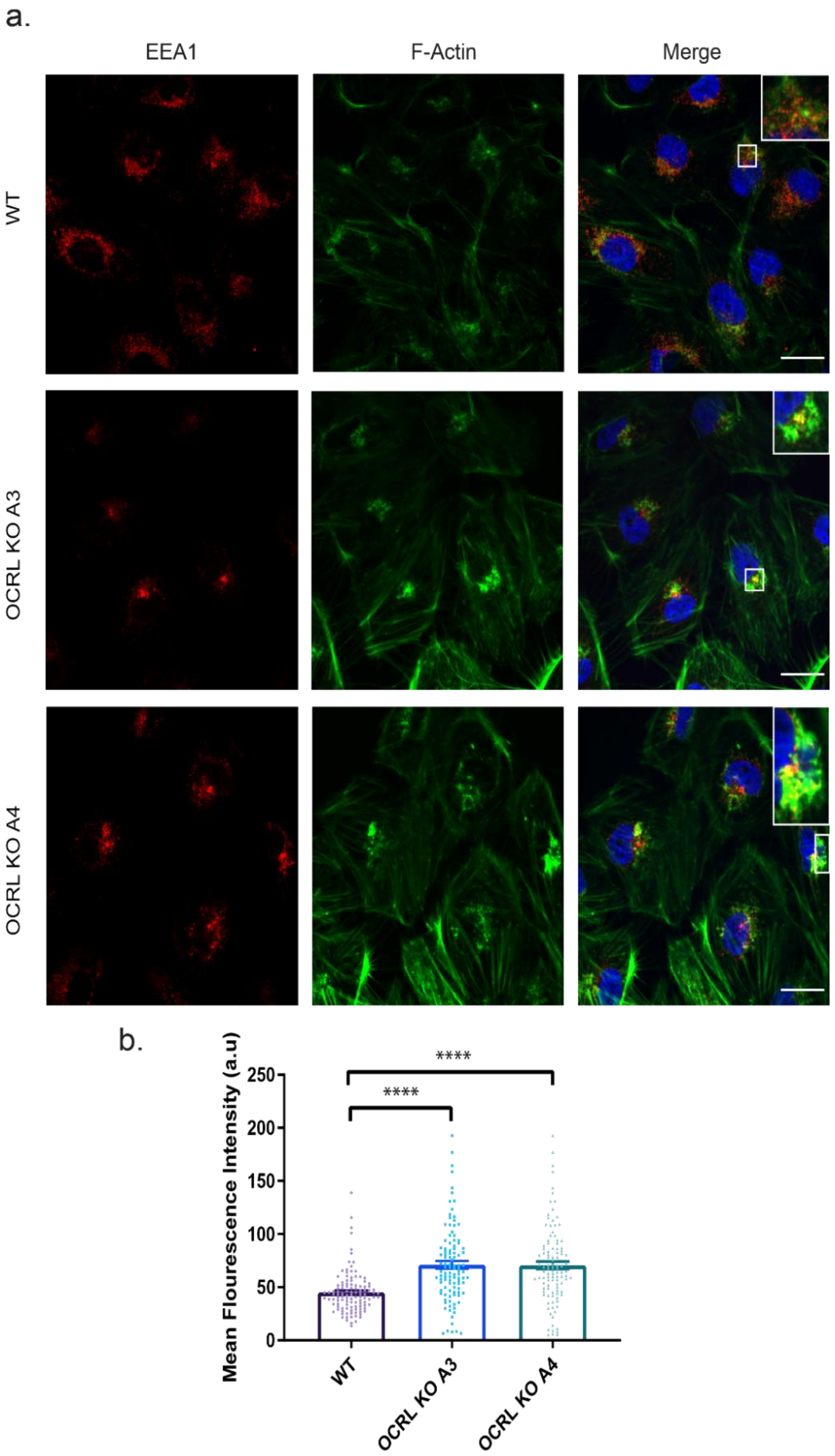

Figure S4. F-Actin staining on Endosomes: (**a**) Immunofluorescence images showing F-actin accumulation in the peri-nuclear region where endosomes are mostly localised. Early endosomes were stained using EEA1 antibody and F-actin stained with Phalloidin fluorescently conjugated with Alexa Fluor 488. Scale bar: 20µm. (**b**) Graph showing increased mean fluorescence intensity of F-actin accumulated on endosomes in OCRL absent cells. The ROI's were chosen based on endosomes and fluorescence intensity was measured in the F-actin channel using ImageJ software. Scatter dot plot with ordinary one-way ANOVA and Dunnett's multiple comparison test, $N = 3$, *, $p < 0.05$; **, $p < 0.01$; ***, $p < 0.001$; ****, $p < 0.0001$, error bar represents mean with ± s.e.m.

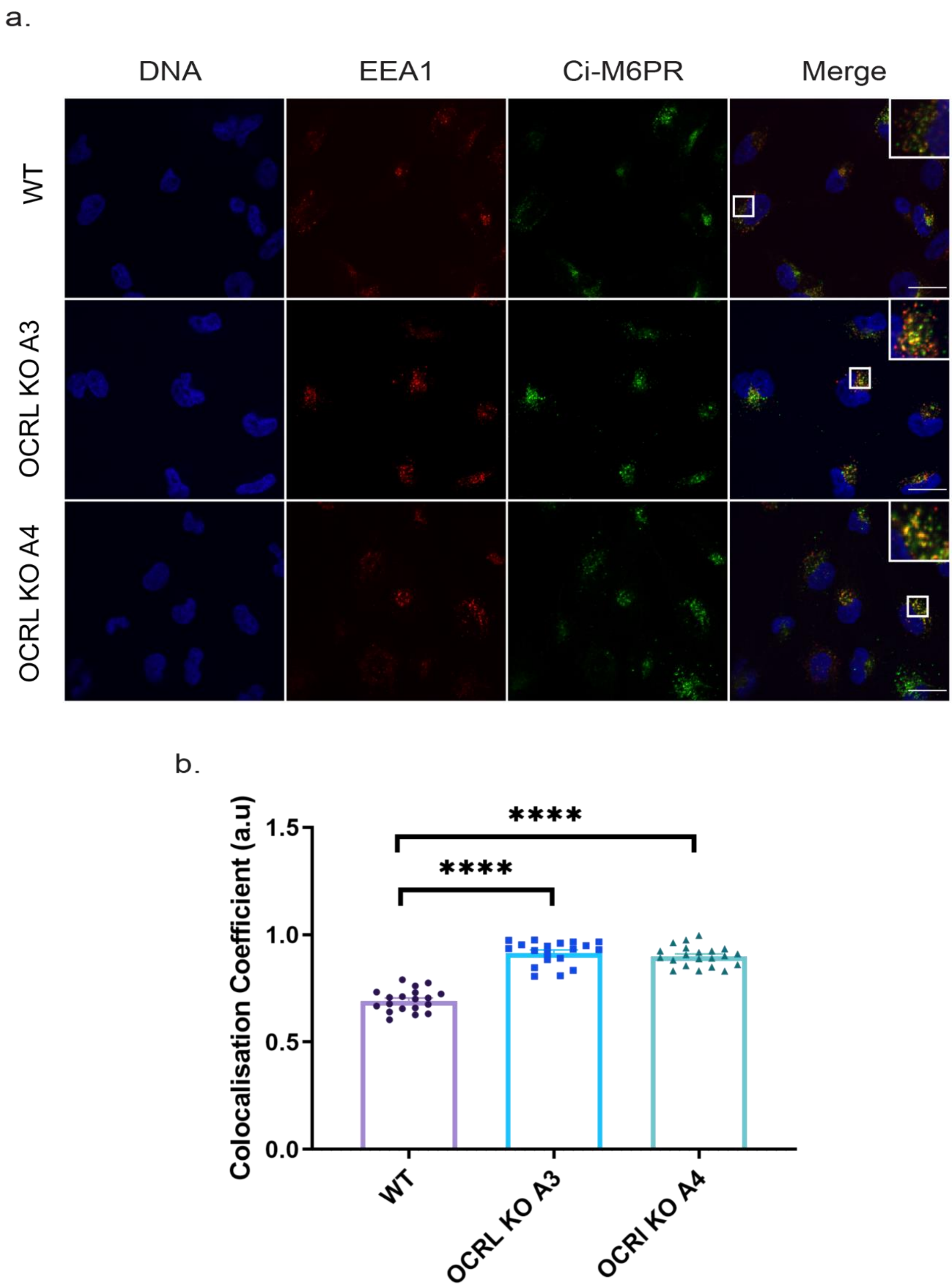

Figure S5. Transport defect from Endosomes to TGN: (**a**) Immunofluorescence analysis of CI-M6PR transport from endosomes to TGN in HK-2 WT and OCRL KO (A3 and A4) cell lines. The cells were fixed and stained with EEA1 and CI-M6PR antibodies. Images were taken by spinning disk confocal microscope, Scale bars: 20μm. Insets show CI-M6PR (green) and EEA1 (red). (**b**) Quantitative analysis of co-localisation using JACoP tool in ImageJ software and represented as column graph with individual values. Ordinary one-way ANOVA and Dunnett's multiple comparison test, $N = 3$ (>50 cells per N), *, $p < 0.05$; **, $p < 0.01$; ***, $p < 0.001$; ****, $p < 0.0001$, error bar represents mean with ± s.e.m.
